# Supplementary material for: Removal of an Azo Dye from Wastewater through the Use of Two Technologies: Magnetic Cyclodextrin Polymers and Pulsed Light
Source: Int J Mol Sci. 2022 Jul 29;23(15):8406. doi: 10.3390/ijms23158406 (PMC9369244; doi:10.3390/ijms23158406)
Supplement: Supplementary file 1 [file ijms-23-08406-s001.zip › ijms-1807718-supplementary.pdf]

SUPPLEMENTARY MATERIAL

Removal of an azo dye from wastewater through the use of two technologies: magnetic cyclodextrin polymers and pulsed light

María Isabel Rodríguez-López [1], José Antonio Pellicer [1], Teresa Gómez-Morte[1], David Auñon[1], Vicente Manuel Gómez-López[1], María José Yáñez-Gascón[1], Ángel Gil-Izquierdo[2], José Pedro Cerón-Carrasco[1], Grégorio Crini[3], Estrella Núñez-Delicado[1], and José Antonio Gabaldón [1,*]

[1] Molecular Recognition and Encapsulation Research Group (REM), Health Sciences Department, Universidad Católica de Murcia (UCAM), Campus de los Jerónimos 135, Guadalupe, E-30107, Spain
[2] Research Group on Quality, Safety and Bioactivity of Plant Foods. Department of Food Science and Technology, CEBAS-CSIC, University Campus of Espinardo - Edif. 25, E-30100 Espinardo, Spain.
[3] Laboratoire Chrono-environnement, UMR 6249, UFR Sciences et Techniques, Université Bourgogne Franche-Comté, 16 route de Gray, 25000 Besançon, France.

Corresponding author: **jagabaldon@ucam.edu,** Phone: #34 968-278622

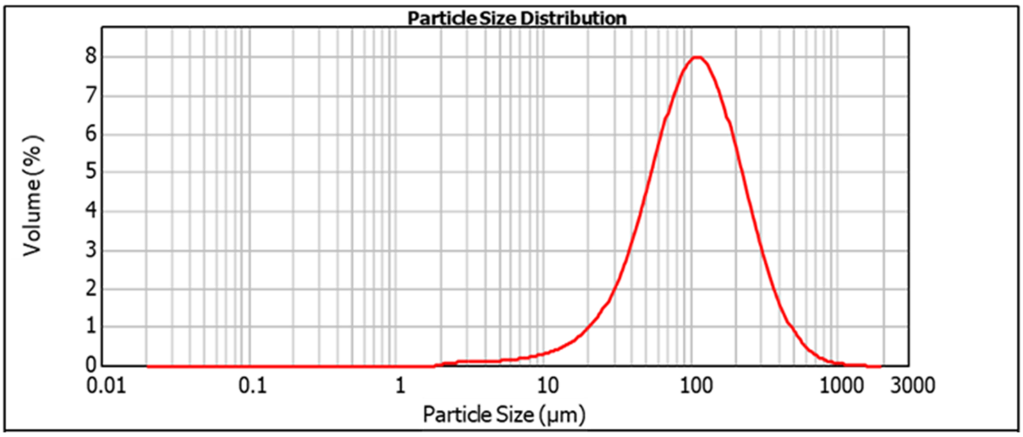

Figure S1 (A). Particle size distribution of β-cyclodextrin-epichlorohydrin polymer

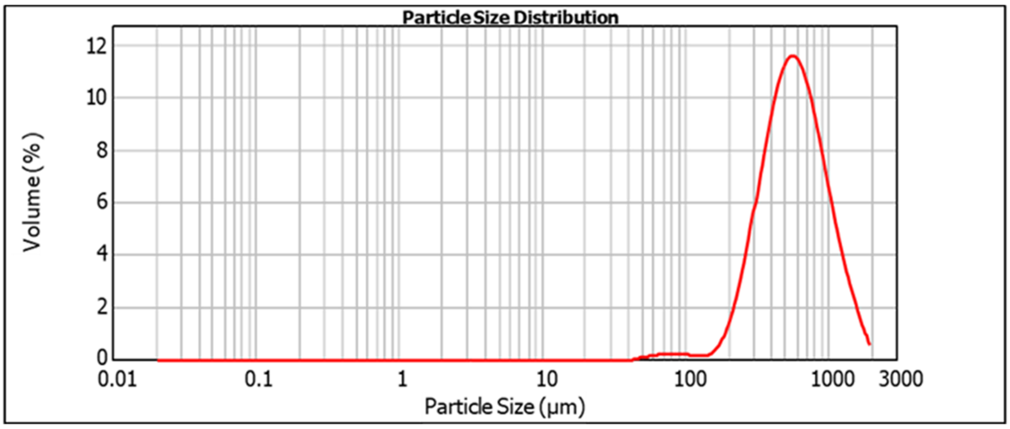

Figure S1 (B). Particle size distribution of β-CDs-EPI polymer-modified Fe_3O_4 nanoparticles

EDX spectra was also performed (Figures S3A and S3B) to determine the iron-oxygen ratio in order to propose a molecular formula.

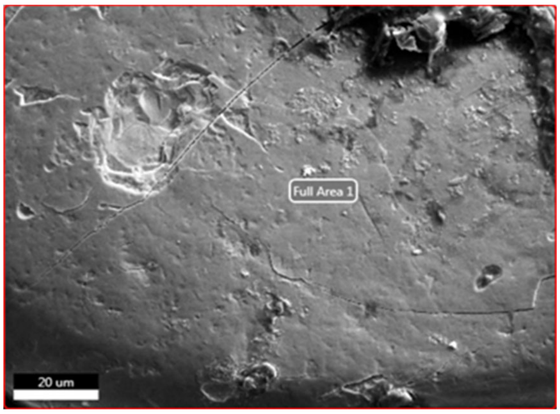

Figure S2 (A). Selected area the β-CDs-EPI polymer-modified Fe_3O_4 nanoparticles to perform EDX analysis (20 kV).

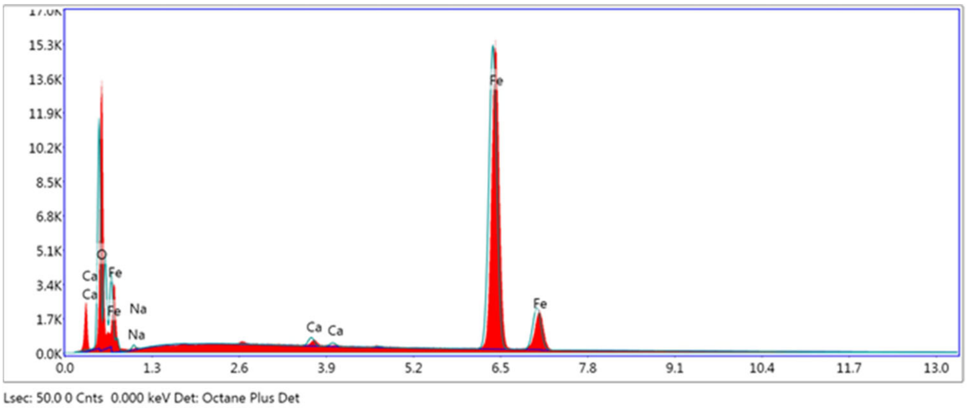

Figure S2 (B). EDX spectra of the β-CDs-EPI polymer-modified Fe_3O_4 nanoparticles selected area (20 kV).

Percentage of selected atoms obtained by EDX analysis

Element	Weight %	Atomic %	Net Int.	Error %	Kratio	Z	R	A	F
O K	17.22	41.55	2,344.16	6.45	0.10	1.21	0.89	0.48	1
NaK	1.03	1.73	56.90	14.71	0.00	1.10	0.92	0.15	1
CaK	0.76	0.73	179.55	6.21	0.01	1.05	0.99	0.93	1.1
FeK	80.99	55.99	8,468.54	1.56	0.77	0.95	1.02	1.0	1

Discarding impurities at 80:20 by weight ratio could be inferred from data described above (Percentage of selected atoms), for iron and oxygen from medium size particles in the polymer. Together with the information provided by elemental analysis in which a 25.26% of carbon and 3.88% of hydrogen was obtained, and considering the polymer constituted only by these atoms plus iron and oxygen, that would mean a 56.68% and 14.17% for the last two elements. Taken into account molecular weight of each of them, this would result in a $(C_{14}H_{22}Fe_7O_6)n$ formula.

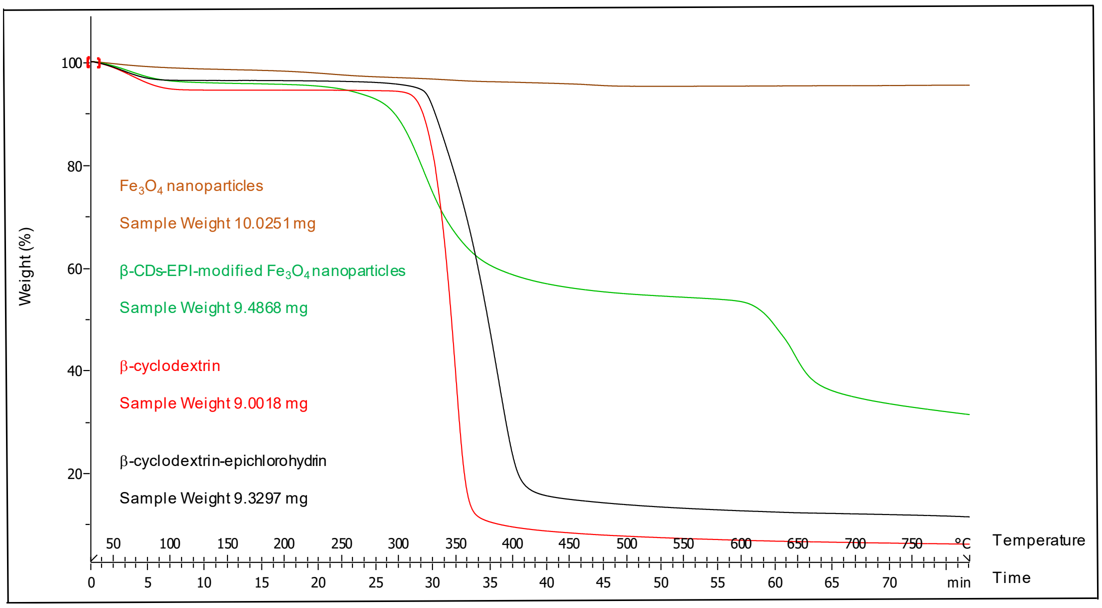

Figure S3. Thermograms (TGA) for the different samples analyzed

A degree of crystallinity between 68-70% was recorded in the material for the independently manufactured Fe_3O_4 nanoparticles and for β-CDs-EPI polymer-modified Fe_3O_4 nanoparticles, corresponding to a diffraction pattern corresponding to a face-centred cubic lattice Fd-3m (227), an inverse spinel structure in which the tetrahedral sites are occupied by Fe^{3+} ions, and the octahedral sites are occupied by an equal number of Fe^{3+} and Fe^{2+} ions.

Comparison with the ICDD (International Centre for Diffraction Data) database showed full equivalence with a magnetite standard sample (PDF 04-006-6551), with no presence at all of crystalline phases with a haematite or maghemite structure.

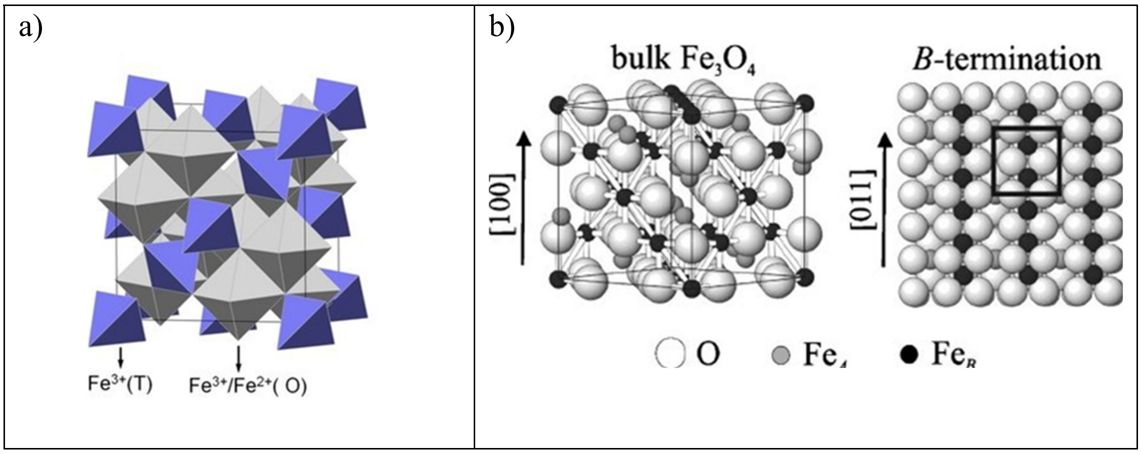

Figure S4. a) Inverse spinel Fe_3O_4 structure in which the tetrahedral sites are occupied by eight Fe3+ ions (blue tetrahedra), and the octahedral sites are occupied by the same number of Fe^{2+} and Fe^{3+} ions (grey octahedra). *Taken from*: Odkhuu D. et al PHYSICAL REVIEW B 98, 094408 (2018). b) Side view of the inverse spinel Fe_3O_4. *Taken from* Fonin M. et al PHYSICAL REVIEW B 72, 104436 (2005).

The pH of the various aqueous solutions of dye at a concentration of 5 × 10^{-5} M, was adjusted using both HCl and NaOH concentrated solutions. UV-Vis absorption spectra were recorded in the 200 - 800 nm range, at a 0.5 nm/s scan rate, using a Shimadzu UV-Vis spectrophotometer mod. 1603.

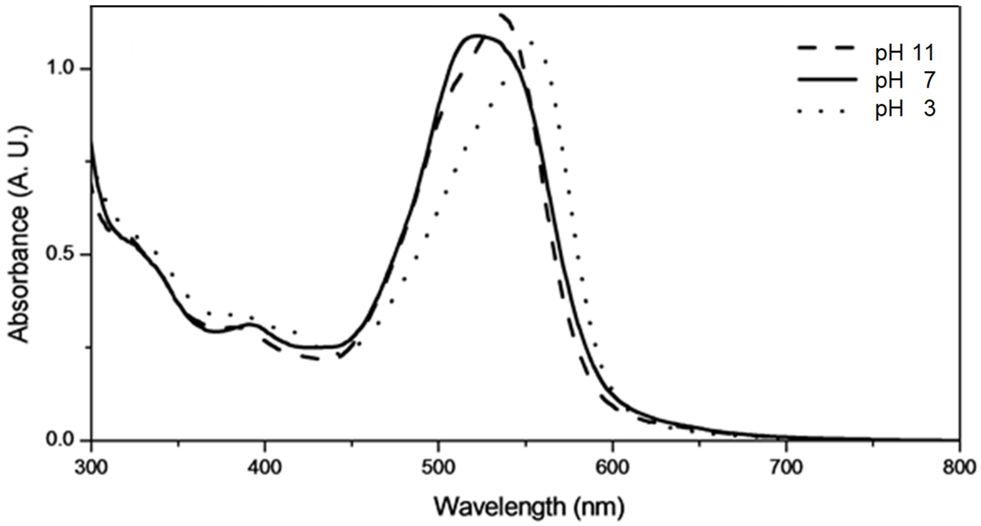

Figure S5. UV-Vis absorption spectra of aqueous solutions of Direct Red 83:1 at different pH.

The absorption spectra are dominated by the classical absorption bands relative to the characteristics chromophore for Direct Red 83:1, result of the interaction between azo functionality (–N=N–) and attached aromatic moieties. In general, there are weaker bands in the UV region attributable to the electronic transitions related to the aromatic rings, while in the visible range there are more intense and wider bands due to $\pi \rightarrow \pi^*$ transitions of donor groups, e.g. an aromatic nucleus containing an auxochromic group such as alkyl side chains, secondary amine, or OH group.

The characteristic absorption bands for Direct Red 83:1 in aqueous solution at pH 7 are located at 527 nm, and the peak position changes varying the pH of aqueous solution for acid media, where the absorption band shifts to 553 nm, and for basic pH is located at 535 nm.

Table S1. Computed interactions energies (in kcal/mol) of the Direct Red 83:1 at several pH values. The predicted protonation state is also reported.

<table>
<tr><th>pH</th><th>charge</th><th>Interaction energy</th></tr>
<tr><td rowspan="2">3</td><td>– 3</td><td>– 43.28</td></tr>
<tr><td rowspan="2">– 4</td><td rowspan="2">– 41.84</td></tr>
<tr><td>5
7</td></tr>
<tr><td rowspan="2">9</td><td>– 5</td><td>– 35.85</td></tr>
<tr><td>– 6</td><td>– 32.55</td></tr>
<tr><td rowspan="2">11</td><td>– 7</td><td>– 30.33</td></tr>
<tr><td>– 8</td><td>– 14.26</td></tr>
</table>

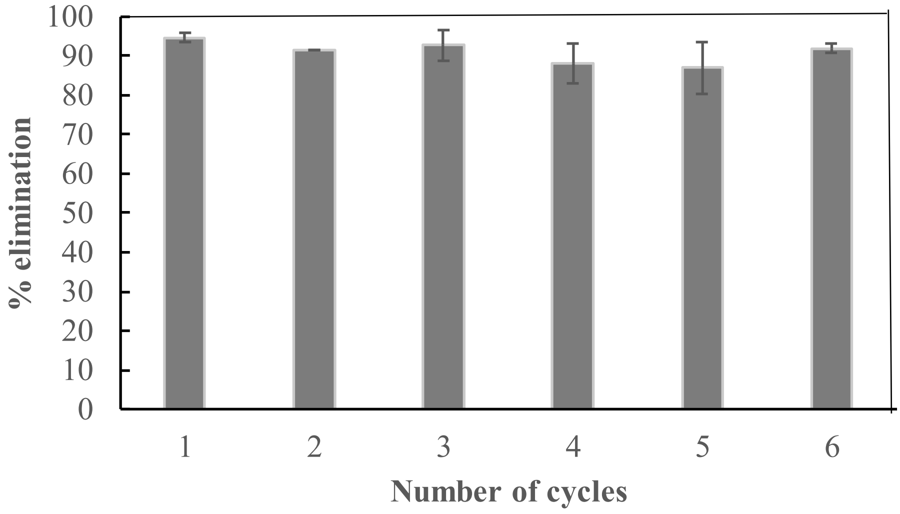

Figure S6. Reusability of the EPI-β-CDs-Fe polymer tested with Direct Red 83:1.
